# The Parentage of British Medical Men of Eminence

**Published:** 1909-06-26

**Authors:** 


### THE PARENTAGE OF BRITISH MEDICAL MEN OF EMINENCE.

In this analytical age interest attaches to every circumstance of greatness, and especially, of course, of intellectual eminence. The following brief and unpretentious survey of the parentage of some of the more distinguished medical men of the past in this country may, therefore, be not without interest, even though it should warrant no very definite conclusions. It cannot escape notice, however, that those drawn from what are conventionally known as the upper classes are very few. Sydenham's father was a country gentleman, Bright the son of a banker at Bristol; while, although Heberden was supposed to come of an old family—rather an ambiguous expression—the exact social status of his parents does not seem to be generally known. It is recorded of Colles that his family was an ancient English one of good means settled in Ireland. After these, names of distinction in medicine are hard to find in this class. One of the professions, on the contrary—namely, the Church—has contributed largely to the number of those who in medicine have benefited posterity as well as served their own generation. Astley Cooper, Liston, Charles Bell, Jenner, and Benjamin Brodie were sons of clergymen of the Established Church of England or Scotland. The father of Mead, a clergyman of the Anglican Church, afterwards took to Nonconformity, while from a minister of the latter body was also descended Morton, author of "Phthisiologia." Graves was the son of a Dublin professor of divinity. Todd's career forms one of the very few instances (although doctors' sons have succeeded well in other walks of life) of a distinguished medical father having a more distinguished medical son. Huxley's father was a schoolmaster. Trade and commerce of very various descriptions have had a place in the early surroundings of many great physicians. Every medical man knows that John Hunter at one time worked for a relative who followed the occupation of cabinet maker. Gull's father was a master wharfinger, Paget's a brewer and shipowner, and Abernethy's a London merchant. Lettsom frankly called himself "a volatile creole," which is the more remarkable since on the European side he was of Quaker origin; he was born in the West Indies. The mixture of races as a source of talent and genius has recently been raised to the position of a serious scientific theory, but there are few other quite definite instances to be found in the history of British medicine. Wollaston, who was attracted mainly by the scientific side of his profession, came of an old and intellectually distinguished Staffordshire family, whose fortune, however, was made in trade in London. Lastly, there are those who were certainly men of the people. Probably this class, if the truth were known, would be considerably larger, for even scientific eminence does not exclude the possibility of pronounced "descent-psychosis"—abnormal pride of ancestry. Willis, the anatomist, was the son of a farmer, Addison of a country grocer, Pott of a London scrivener, Huxham, an original observer, and deviser of the pharmacopœial compound tincture of cinchona, of a butcher. The most illustrious Harvey sprang from Kentish yeoman stock.

